# Assessment of safety and immunogenicity of a protein subunit COVID-19 vaccine (ABDALA) in pregnant women: a single-cohort, multicenter, observational ESPIRTA study

**DOI:** 10.1186/s12884-026-09488-1

**Published:** 2026-06-24

**Authors:** Jorge Antonio Aguilar Estrada, Idania Baladrón Castrillo, Ivan Campa Legrá, Pablo Arsenio Díaz Reyes, Maria Antonieta Costafreda Blanco, Yudith Cambrero Martínez, Yuleisi de la Caridad Preval Calsado, Bárbara Enríquez Domínguez, Osmany Franco Argote, Yoisi Verrier Quesada, Yaima León Molina, Miguel Sarduy Nápoles, Kenia Almenares Rodríguez, Ernesto Rodríguez Martínez, Rolando Selgas Lizano, Jesús E. Sánchez García, Marel Alonso Valdés, Gilda Lemos Pérez, Yudisay Reyes Pelier, Gabriel Padrón Palomares, Miladys Limonta-Fernández, Marta Ayala-Avila, Liz Alvarez-Lajonchere Ponce de León, Verena L. Muzio- González, Espirta Research Group

**Affiliations:** 1 Ramón González Coro” Maternal Hospital, Havana, Cuba; 2 Genetic Engineering and Biotechnology Centre (CIGB), P.O. Box 6162, Havana, Cuba; 3 Faculty of Medical Sciences, Salvador Allende, Havana, Cuba; 4 Eusebio Hernandez” Maternal Hospital, Havana, Cuba; 5 Angel Arturo Aballí” Maternal Hospital, Havana, Cuba; 6 Hijas de Galicia” Maternal Hospital, Havana, Cuba; 7 America Arias” Maternal Hospital, Havana, Cuba; 8 Cybernetics, Mathematics and Physics Institute, Havana, Cuba

**Keywords:** COVID-19, SARS-CoV-2, Vaccine, Pregnant women, Newborn

## Abstract

**Background:**

Pregnant and lactating women are generally excluded from clinical trials during vaccine development. While the safety, inmunogenicity, efficacy, and effectiveness of the Abdala vaccine against COVID-19 have been demonstrated in the general population, there is a lack of specific information regarding its benefits for pregnant women. Consequently, this study was undertaken to evaluate the safety of the Abdala vaccine in pregnant women and their newborns, as well as to assess the maternal immune response elicited by the vaccination and its capacity for passive immunity transfer to the newborn.

**Methods:**

A simple cohort observational multicenter study was conducted across five maternity hospital in Havana City, Cuba. A hybrid approach was employed, integrating both retrospective and prospective data collection methods. The study analyzed all events occurring from the first dose of the Abdala vaccine during pregnancy, delivery, and the postpartum period, as well as those related to the fetus-/neonate. To provide contextual data from our settings as a reference for descriptive analyses, statistical information concerning some pregnancy-related and fetal-neonatal events from the same five maternity hospitals in the year 2020 was utilized (historical data). Immunogenicity analyses were conducted in a subgroup of participants from a single maternity hospital, measuring antibodies against the receptor-binding domain of SARS-CoV-2 (Anti-RBD IgG antibodies) and neutralizing antibodies (Nab) against two SARS-CoV-2 strains (D614G and Omicron B.1.1.529) were measured in both maternal and umbilical cord sera. Additionally, anti-IgA antibodies were evaluated in a colostrum samples. Antibody transfer across the placenta and breast milk was also analyzed. Various comparisons were made regarding gestational age at birth, vaccination trimester, timing from vaccination to delivery, and receipt of a booster dose, among other analyses. A formal sample size estimate was not made. Pregnant women who attended the aforementioned hospitals and met the established criteria were included in the study cohort. Descriptive statistics were utilized to characterize the study population. The Wilcoxon sum rank test was used for most immunological evaluations, while logistic regression analyses estimated the effects of different variables. The correlation between anti-RBD IgG titers in maternal and umbilical cord sera was assessed using Pearson's correlation coefficient. All statistical tests were performed at a significance level of *p* < 0.05.

**Results:**

The study was conducted in five Cuban hospitals from December 2021 to June 2022, involving a total of 940 pregnant women women who received the Abdala vaccine during their pregnancy. The common adverse events reported within the 72 h post-vaccination with the Abdala vaccine were consistent with previous findings using this vaccine in clinical trials and widespread vaccination campaigns in the general population, predominantly presenting as pain at the injection site (4.9%), somnolence (2.6%), and headache (2.3%). All reported events were of mild intensity. In terms of maternal morbidity, the predominant event noted was SARS-CoV-2 infection, with 83 cases (8.83%), primarily categorized as asymptomatic cases or exhibiting mild symptomatic disease. Overall, IgA titers were detected in 202 colostrum samples, with GMT of 1,227 (95% CI 986; 1,527). High anti-RBD IgG titers were found in 189 maternal and 231 umbilical cord blood samples, with GMT of 1,392.15 (95% CI 1,174; 1,651) and 1,923 (95% CI 1,625; 2,275) respectively. The placental transfer ratio (PTR) of anti-RBD IgG titers had a median of 1.54 (IQR 1.48), indicating effective transfer. The PTR of NAb exceeded 1 for both D614G and Omicron (B.1.1.529), being significantly higher in full-term newborns compared to premature newborns.

**Conclusions:**

The ESPIRTA study provides valuable information concerning the application of the Abdala vaccine in specific populations, such as pregnant women, which was not available prior to this study. The safety evaluation of the Abdala vaccine during pregnancy, delivery and the puerperium, as well as in fetus-newborn, revealed no safety signals, as indicated by this cohort study. Elevated anti-RBD IgG titers were detected, in both in maternal serum and cord samples, indicating a positive correlation between them. Moreover, an efficient transfer of IgG antibodies across the placenta was demonstrated. The high anti-IgA titers found in the colostrum may provide an additional advantage regarding the passive transfer of antibodies from mother to newborn through breastfeeding. Furthermore, neutralizing antibodies against two SARS-CoV-2 strains, D614 G and the more recent Omicron B.1.1.529 variant, were identified. Further research is recommended to assess the long-term safety and efficacy of the Abdala vaccine for pregnant women and their newborns.

**Supplementary Information:**

The online version contains supplementary material available at https://doi.org/10.1186/s12884-026-09488-1.

## Introduction

The COVID-19 pandemic has posed unprecedented challenges to global public health, particularly impacting vulnerable populations, including pregnant women. Initially excluded from vaccine clinical trials, pregnant women found themselves in a position of heightened risk of SARS-CoV-2 infection and its complications.

Although no significant differences in maternal and perinatal outcomes were found between pregnant women with and without COVID-19 [1], accumulating evidence has shown that pregnant women have an increased risk of developing severe forms of COVID-19 compared with non-pregnant women of the same age [1–3]. This has led to a growing consensus on the necessity of vaccination for this population group. The Latin American Federation of Obstetrics and Gynecology Societies, the International Federation of Gynecology and Obstetrics and the American College of Obstetricians and Gynecologists recommended prioritizing access to vaccination for pregnant or lactating women and ensuring proper follow-up for vaccinated pregnant women and their offspring [4–6]. Newborns receive passive immunity through antibodies transferred via the placenta and breast milk following either natural infection or vaccination. Thus, maternal vaccination against COVID-19 has the potential not only to protect the pregnant woman but also to confer fetal and neonatal benefits, providing immunity to newborns [7, 8].

The Cuban’s COVID-19 Abdala is a recombinant subunit vaccine, developed at the Centre for Genetic Engineering and Biotechnology (CIGB), based on the receptor-binding domain (RBD) of the SARS-CoV-2 spike protein, produced in *Pichia pastoris* yeast and adjuvant to alumina [9]. The Abdala vaccine has demonstrated to be safe, highly immunogenic and efficacious in adults, children and adolescents [10–14]. Following its Emergency Use Authorization (EUA), granted by the Cuban Center for State Control of Medicines and Medical Devices (CECMED) on July 9, 2021, massive vaccination campaigns were carried out in the country. However, specific data regarding its administration to pregnant women were not available. Considering the complex epidemiological situation in the country, with a notable increase in COVID-19 morbidity and mortality since mid-2021, the Cuban Ministry of Public Health (MINSAP) implemented a health intervention to vaccinate pregnant women in July–August of that same year, using the Abdala vaccine, which was the only authorized anti-COVID-19 vaccine available in the country at that time.

Given the limited data from clinical trials concerning the safety and efficacy of Abdala vaccine in pregnant women, it became necessary to undertake a post-licensing observational study to gather information on adverse events and to assess the immunogenicity in both mothers and their newborns. The present study, designated as ESPIRTA, was conducted with the aim to assess the safety of Abdala vaccine in pregnant women and their newborns, as well as to evaluate its capacity to induce an immune response that can be efficiently transmitted to the fetus via the placenta and to the newborn through breastfeeding. This study is presented in accordance with the Strengthening the Reporting of Observational Studies in Epidemiology (STROBE) reporting guideline.

## Methods

### Study design

A multicenter observational study with a single cohort of pregnant women previously immunized against COVID-19 with Abdala vaccine, whose delivery occurred between December 2021 and June 2022 at five maternity hospitals in Havana City, Cuba. The participating institutions were: “Ramón González Coro”, “Eusebio Hernandez”, “Angel Arturo Aballí”, “Hijas de Galicia” and “America Arias” hospitals. The study was conducted by clinical researchers who selected the study participants based on established inclusion and exclusion criteria outlined in the investigational protocol. These researchers, who were specialists in gynecology and obstetrics, were also responsible for conducting clinical evaluations and safety assessments, as well as collecting the primary data generated throughout the study. They were mere observers without exerting any influence on the exposure factor of vaccination against SARS-CoV-2 with Abdala vaccine, which had already been administered prior to participant inclusion.

Given that exposure to the Abdala vaccine occurred several months before the inclusion of the participants in the study, a hybrid approach was employed, combining retrospective and prospective data collection methods, to offer a more accurate assessment of study outcomes. Adverse events following immunization as well as all events that occurred during pregnancy were documented retrospectively. Events occurring during delivery, postpartum, and in newborns were identified and recorded prospectively. The assessment of the immunogenicity was carried on maternal sera, umbilical cord and colostrum samples, collected exclusively from participants at the “Ramón González Coro” maternity hospital. Prior to the initiation of the study, researchers received training on the informed consent process, adherence to the protocol, completion of case report forms, and the collection, labeling, and preservation of samples. On-site monitoring by the sponsor’s monitors ensured compliance with these processes and confirmed the accuracy of all case report forms. The study received approval from the Ethics Committees of the participating medical institutions as well as from CECMED.

### Participants

The study population comprised pregnant women who attended the participating hospitals and had received the Abdala vaccine as part of a health intervention implemented by MINSAP six months earlier (July–August 2021). Pregnant women were enrolled based on specific inclusion criteria: those who had received three doses of the primary vaccination with the Abdala vaccine (50 µg RBD, in a 0.5 mL volume, administered intramuscularly in the deltoid area at 0, 14, and 28 days) either during pregnancy or prior to conception (within the teratogenic window, defined as up to 30 days before the date of last menstrual period). Participants, with or without booster doses, provided written informed consent for participation in the study, which allowed for access to their medical records and the collection of maternal blood, cord blood samples, and colostrum, to evaluate immunogenicity in both the mother and her newborn. The exclusion criteria were: pregnant women who were unable to provide informed consent, prior immunization with other COVID-19 vaccine, those who were participating in other research or drug studies, or received any investigational product during pregnancy, and those with incomplete medical records, vaccination or obstetric cards.

### Procedures

Demographic and baseline characteristics of the pregnant women at the time of inclusion were collected from the hospital medical records of the participants.

To assess the safety of the Abdala vaccine within this cohort of pregnant women, records of all adverse events that occurred after the administration of any dose of the primary immunization schedule or booster dose were gathered. An adverse event following immunization (AEFI) was defined as any untoward medical occurrence following immunization which does not necessarily have a causal relationship to the vaccine. The adverse event may be any unfavorable or unintended sign, abnormal laboratory finding, symptom or disease. Safety information was organized in two sets, taking into account that the collection of adverse events spanned from the administration of the first vaccine dose throughout pregnancy, delivery and the puerperium as well as neonatal events. A hybrid approach, combining both retrospective and prospective data collection techniques, was employed.

The first set included the evaluation of AEFI that occurred within the first 72 h post-vaccination. This information was collected retrospectively. The specified timeframe enabled the identification of the most common events that have a close temporally relationship with vaccination, including injection site reactions, systemic reactions and other medical events following vaccination. The vaccination status was documented using the vaccination card provided during the vaccination campaign, which contained the information related to COVID-19 vaccination: the specific vaccine administered, the dates of each immunization, batch number, and details concerning the booster dose. Information regarding AEFI was collected from medical records of the participants, within the initial 72 h following each vaccination. According the frequency of occurrence (in percentage) AEFI were classified into five categories following the World Health Organization (WHO) recommendations: very common ≥ 10%, common (frequent) ≥ 1% and < 10%, uncommon (infrequent) ≥ 0.1% and < 1%, rare ≥ 0.01% and < 0.1% and very rare < 0.01% [15]. All events that occurred during pregnancy were also retrospectively gathered from the obstetric cards and medical records of the participants. The second set comprised events that occurred during delivery or the puerperium, along with neonatal events. These events were identified and collected prospectively, once the pregnant women were included in the study. This information was also documented in the participants' medical records.

Given that ESPIRTA was an observational multicenter single cohort study without a concurrent unvaccinated group, we utilized historical data derived from hospital statistics at the five participating sites in the ESPIRTA study for the year 2020 as a reference. This period was selected as an appropriate reference for historical data because it precedes the notable morbidity and mortality rates among pregnant women attributed to the pandemic observed in the first half of 2021, and prior to the initiation of mass vaccination campaign targeting pregnant women. These considerations were important in minimizing biases in the analyses related to the pandemic and COVID-19 vaccination.

Immunogenicity analyses were performed on a subgroup of participants from a single maternity hospital, out of the five institutions involved in the study. This approach was outlined in the research protocol due to logistical reasons arising from the pandemic context in which the study was conducted. The decision aimed to facilitate a more affordable assessment of immunogenicity across various biological samples and laboratory tests. Consequently, samples of colostrum, cord blood and maternal blood were collected exclusively from pregnant women and newborns at the "Ramón González Coro" Maternal Hospital, the main investigational site in the study.

Anti-RBD IgG antibodies were quantified by the UMELISA SARS-CoV-2 ANTI-RBD test (Immunoassay Centre, Havana), approved by CECMED [16]. Titers were given in Binding Antibody Units per milliliter (BAU/mL) with a cut-off value for positivity of 25 BAU/mL. In colostrum samples a non-commercial ELISA test developed at CIGB was used to determine anti- IgA titers expressed in arbitrary units per milliliter (AU/mL). For each biological sample, two independent dilutions were prepared and measured as technical replicates. Neutralizing antibody (Nab) titers were detected by a standard virus micro neutralization assay using infective SARS-CoV-2 (D614G and Omicron B.1.1.529 viral strains) carried out at Civilian Defense Scientific Research Centre, Cuba. Viral neutralizing titers were calculated as the highest serum dilution with an optical density (OD) higher than the cut-off value. Results were given in Nab titers ID50 [17]. Placental transfer ratio (PTR) was calculated as the IgG concentration in umbilical cord blood divided by the maternal IgG concentration [18].

### Outcomes

The main outcomes were safety (by the evaluation of all adverse events following immunization, during pregnancy, delivery and the puerperium, in addition to neonatal events) and immunogenicity (by the evaluation of IgG anti-RBD antibodies in maternal serum and umbilical cord blood, anti-IgA in colostrum, neutralizing antibodies against two variant strains of SARS-CoV-2 in both maternal and umbilical cord blood and assessing placental antibody transfer).

### Statistical analysis

A formal sample size estimate was not made. The study cohort consisted of pregnant women who attended the aforementioned hospitals and fulfilled the established criteria. Descriptive statistics were employed to characterize the study population. Quantitative data were presented as mean ± standard deviation or median with interquartile range (IQR), while qualitative data were expressed as frequencies (%). For the analysis of the immune response, the geometric mean of anti-RBD IgG titers (GMT) in maternal and cord blood, as well as anti-IgA antibodies in colostrum, were calculated as the arithmetic mean of logarithmically transformed titers, followed by an inverse transformation. Comparisons of anti-RBD IgG and anti-IgA in colostrum were evaluated by Wilcoxon sum rank test. The correlation between anti-RBD IgG titers in maternal and umbilical cord sera was estimated using Pearson's correlation coefficient. Neutralizing Ab concentrations were compared using the Wilcoxon test for related samples. The placental transfer ratio (PTR) was calculated by dividing the concentration of cord IgG by the concentration of maternal IgG antibodies. Derived from it, a smoothed curve analysis was performed to determine the relationship between the mean placental transferratio and the timing of delivery at each gestational age, with comparisons made according to vaccination trimester using the one-way ANOVA test.

A logistic regression model was used to evaluate the influence of demographic and baseline variables on risk of symptomatic disease occurring 14 days post-completion of the three-dose immunization schedule. Additionally, logistic regression analyses were conducted to evaluate the effect of the time interval from the last vaccine dose to delivery (where immunogenic assessment was carried out) and PTR ≥ 1. Subgroup analyses were done, taking into account whether or not a booster dose was administered. The results were presented in terms of Odds ratios (OR) along with the 95% confidence intervals (CI) and p values. Furthermore, a logistic regression model using the backward selection procedure with the AKAIKE Information Criterion (AIC) was used to examine the influence on the immune response of control variables and adverse events encountered throughout gestation, delivery, and the puerperium, as well as those observed in the offspring. AIC was used to compare different possible models to ascertain the most suitable fit for the data.

All statistical tests were performed at a significance level of *p* < 0.05 and processed in the R 4.1.2 programming environment and SPSS version 26 software. Statistical analyses were conducted by independent statisticians at the Cybernetics, Mathematics, and Physics Institute (ICIMAF) in Havana.

### Role of the funding source

The funder of the study had a role in study design, data interpretation, and writing of the report, but had no role in data collection or data analysis.

## Results

### Characteristics of the study population

**Fig. 1:**
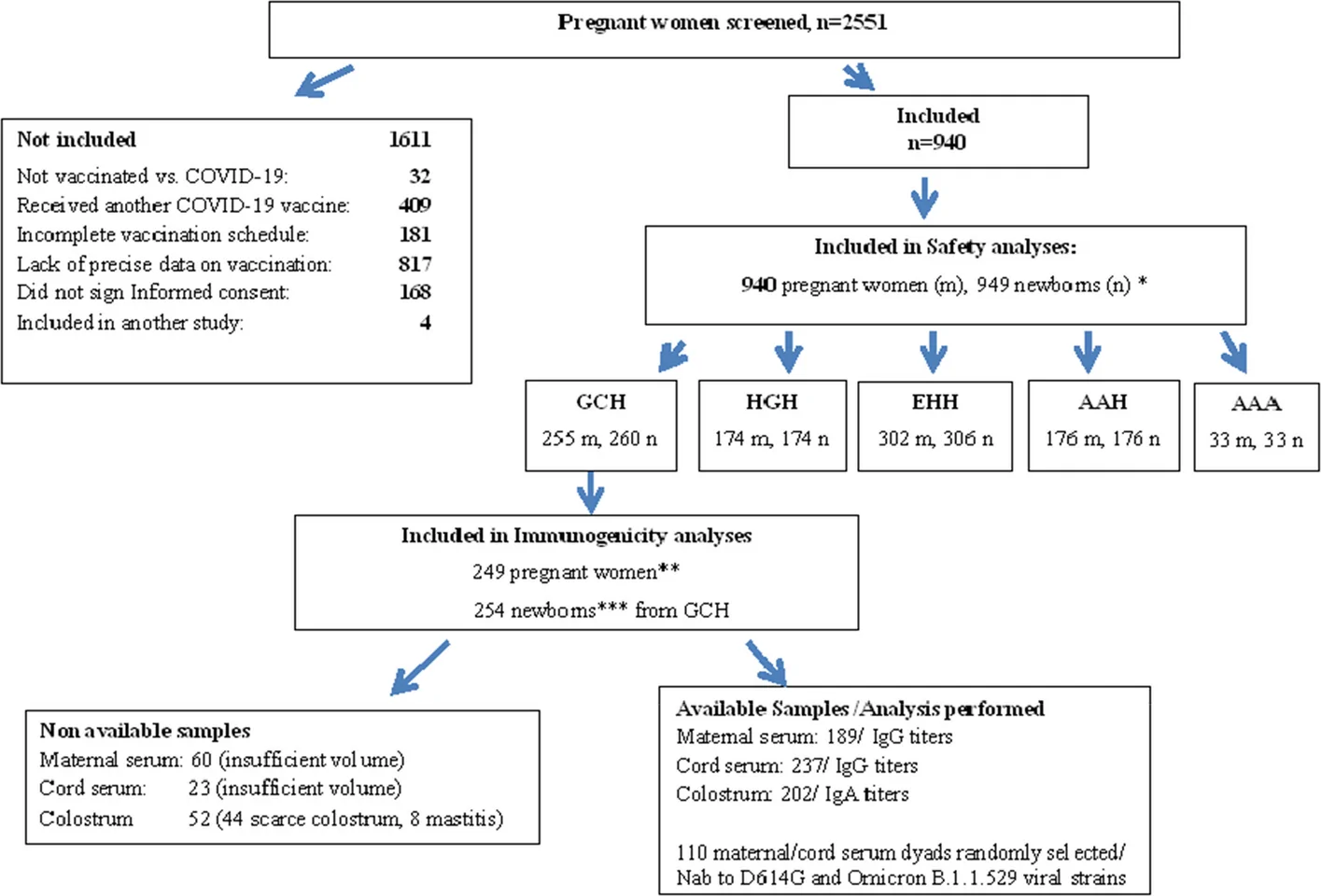
Study flow chart. Legend: * 949 newborns were included as a result of 8 multiple gestations in (7 twins and 1 triplet) from the 940 pregnant women included, GCH: "Ramón González Coro" Maternal Hospital, HGH: “Hijas de Galicia” Maternal Hospital, EHH: “Eusebio Hernandez” Maternal Hospital, AAH: “America Arias” Maternal Hospital and AAA: “Angel Arturo Aballí” Maternal Hospital, Havana, Cuba. ** referred to 249 out of 254 pregnant women included in the immunogenicity evaluation in GHC (no serum was collected in 6 participants), *** referred to 254 out of 260 newborns included in that evaluation (no umbilical cord sample was collected in 6 participants)

From December 23th 2021 to June 9th 2022, a total of 940 pregnant women were enrolled from an initial screening of 2,551 individuals. Their disposition is shown in Fig. 1. The data indicate that 1,611 out of 2, 551 screened individuals were not included in the study, representing 63.2% of the total. The main reason for exclusion was the absence of the vaccination card affecting 817 out of 1,611 not included women, which accounts for 50.71%. This absence obstructed the verification of the completion of the three-dose Abdala vaccine immunization schedule, including vaccination dates and batch numbers. Additionally, factors such as lack of COVID-19 vaccination, prior administration of other COVID-19 vaccines, and incomplete vaccination schedules accounted for 622 out of 1,611 excluded women, which correspond to 38.61%.

Table 1 show the demographic and baseline characteristics of the participants. The mean (± SD) age of the subjects was 27.0 ± 6.1 years, with a significant majority of 879 women (93.5%), being over the age of 18. Regarding nutritional status, 239 participants (25.4%) were classified as overweight, and 403 (42.9%) were categorized as obese. The most frequently reported comorbidities included bronchial asthma (15.2%) and high blood pressure (10.1%).

**Table 1:** Demographic and baseline clinical variables of the study population

Variables	*N* = 940	
Age	Mean ± SD	27.4 ± 6.1
	Median (IQR)	27.0 (9.0)
	(Min – Max)	(14; 44)
	≤ 18 years	61 (6.5%)
	> 18 years	879 (93.5%)
Body Mass Index (BMI)	Mean ± SD	28.4 ± 5.2
	Median (IQR)	27.8 (6.4)
	(Min – Max)	(15.0; 68.4)
	*Missing data*	3 (0.3%)
Nutritional Condition	BMI < 18.8 (Underweight)	10 (1.1%)
	18.8 ≤ BMI < 25.6 (Normal)	285 (30.3%)
	25.6 ≤ BMI < 28.6 (Overweight)	239 (25.4%)
	BMI ≥ 28.6-(Obese)	403 (42.9%)
	*Missing data*	3 (0.3%)
Ethnicity	White	439 (46.7%)
	Black	193 (20.5%)
	Mestizo	305 (32.4%)
	Asian Cuban*	3 (0.3%)
Substance use	Alcohol	78 (8.3%)
	Coffee	282 (30.0%)
	Smoking	65 (6.9%)
	Ex-smoker	48 (5.1%)
Maternal Comorbidity**	Bronchial asthma	143 (15.2%)
	High blood pressure	95 (10.1%)
	Diabetes Mellitus	22 (2.3%)
	Heart disease	15 (1.6%)
	Hypothyroidism	15 (1.6%)
	Gestational Diabetes	13 (1.4%)
	Epilepsy	9 (1.0%)
	Allergy	8 (0.9%)
	Cancer	5 (0.5%)
	Gestational hypertension	4 (0.4%)
	Chronic gastritis	3 (0.3%)
	Hyperthyroidism	3 (0.3%)
	Thrombophilia	3 (0.3%)
	Myopia	2 (0.2%)
	Spina bifida	2 (0.2%)

*SD* Standard deviation, *IQR* Interquartile rank

^**^recorded comorbidities with a frequency of only 1 case (each one): Arrhythmia, Rheumatoid arthritis, Euthyroid goiter, Cervicitis psoriasis, Vagal crisis, Anosmia, Hip dysplasia, Multiple sclerosis, Scoliosis, Pulmonary stenosis, Atrial extra systole, Acute esophagitis, Genital herpes, Congenital adrenal hyperplasia, Epilepsy, Mild mental retardation, Lichen planus, Venous capillary malformation on left side of face, Mastitis, Pituitary micro adenoma, Migraine, IgA immune nephropathy, NIC-I, Partial paralysis, Psoriasis, Chronic retrovirus, Bipolar disorder, HIV positive

^*^referred to 3 participants from Asian ancestors

Table 2 provides comprehensive data on gestation, delivery, and vaccination status. The mean (± SD) gestational age at delivery was 38.7 ± 2.1 weeks, with 858 deliveries (91.3%) classified as occurring at term. The mean gestational age at vaccination (± SD) was 11.6 ± 8.5 weeks. A majority of women received the vaccine during the first trimester (498 participants, 53.0%) or the second trimester (384 participants, 40.9%). A minor proportion (28 participants, 3.0%) was vaccinated in the pre-conception stage which falls within the teratogenic window, whereas 29 participants (3.1%) were vaccinated during the third trimester. More than half of the participants (502, 53.4%) received a booster dose during their pregnancy. The interval between the last vaccine dose and delivery was 15.4 ± 9.8 weeks.

**Table 2:** Characteristics of gestation, delivery and vaccination

Variables	*N* = 940	
Gestational age at vaccination (weeks)	Mean ± SD	11.6 ± 8.5
	Median (IQR)	12.4 (15.0)
	(Min – Max)	(−4; 38.1)
	*Missing data*	1 (0.1%)
Trimester of pregnancy in which vaccination began	Pre-conceptional*	28 (3.0%)
	I	498 (53.0%)
	II	384 (40.9%)
	III	29 (3.1%)
	*Missing data*	1 (0.1%)
Booster vaccination	Yes	502 (53.4%)
	No	438 (46.5%)
Time between last vaccine dose and delivery (weeks)	Mean ± SD	15.4 ± 9.8
	Median (IQR)	15.0 (12.0)
	(Min – Max)	(1; 43)
	*Missing data*	1 (0.1%)

*SD* Standard deviation, *IQR* Interquartile rank

^*^defined as up to 30 days before the date of last menstrual period

### Safety results

A total of 157 AEFI were reported within the 72 h post-vaccination with Abdala vaccine in only 16.7% of the vaccinated pregnant women. Twenty types of events were documented. Common events, defined as those reported between 1% and up to less than 10% of the total of vaccinated pregnant women, included pain at injection site (46 cases) and systemic events of somnolence (24 cases), headache (22 cases) and fever (12 cases), with a frequency of 4.9%, 2.6%, 2.3% and 1.3% respectively. These four adverse events accounted for the 66.2% of the total events reported (104 out of 157). The remaining events (53 cases) were infrequent, occurring in less than 1% of the vaccinated women and included local events at the injection site such as inflammation (8 cases), induration (2 cases), and burning sensation (4 cases), as well as general discomfort (7 cases), asthenia (5 cases), nausea (3 cases), and vomiting (2 cases), among others.

All reported events were of mild intensity. These were consistent with known events of Abdala vaccine, based on previous clinical trial results and widespread vaccination campaigns in the general population, with a temporal relationship to the vaccinations and no other potential causes identified; thus, they were classified as attributable to vaccination. No serious adverse events were reported.

Out of the 940 pregnant women included in the study, 566 (60.2%) experienced an adverse event related to pregnancy, delivery or the puerperium following vaccination with Abdala. Regarding maternal morbidity, the most reported common events were SARS-CoV-2 infection (83 cases, 8.83%), gestational hypertension (67 cases, 7.13%), gestational diabetes (48 cases, 5.11%) and infections of the urinary tract (45 cases, 4.79%). Among these, SARS-CoV-2 infection was the most reported event contributing to maternal morbidity. In the ESPIRTA study, 857 out of the 940 participants (91.17%) were not infected with SARS-CoV-2, while 83 pregnant women (8.83%) had confirmed infections via RT-PCR at any point after receiving the first dose of the Abdala vaccine. In the frame period of 14 days after the completion of the three-dose immunization schedule, SARS-CoV-2 infection was confirmed in 69 out of 935 participants, representing 7.38% (five women were not considered in the denominator as they as they had been infected prior to receiving the third dose of the schedule). The mean interval between 14 days after completing the three-dose immunization schedule and the onset of symptomatic disease was 44.6 weeks (CI 95% 43.9, 45.3). No SARS-CoV-2 infections were recorded prior to vaccination. A history of symptomatic COVID-19 during pregnancy was found in 69 out of the 83 infected women, accounting for 83.13% of the total infected ones. These patients were hospitalized, in accordance with the COVID-19 protocols established by MINSAP, despite the fact that the majority of them exhibited mild disease, specifically 65, representing 94.2% of all symptomatic cases. Furthermore, only 4 patients (5.79%) experienced moderate disease, while no cases of severe illness were documented. A logistic regression model was used to evaluate the influence of demographic and baseline variables on the risk of symptomatic disease, starting 14 days post-completion of the three-dose immunization schedule. The results indicated that bronchial asthma significantly increased the risk of symptomatic infection, with and odds ratio of 2.10 (95% CI 1.16; 3.80).

Regarding obstetric pathologies, the most frequently reported events included cesarean Sect. (225 cases, 23.94%) and thread of premature birth (34 cases, 3.62%). Regarding pathologies of ovular annexes, premature rupture of membranes was the most frequent event, recorded in 65 cases (6.91%). The most frequent fetal or neonatal events included pre-term birth (72 cases, 7.58%), low birth weight (52 cases, 5.48%) and small for gestational age (38 cases, 5.48%). Stillbirths that occurred at or after 20 weeks were documented in 6 cases, representing a frequency of 0.6%. These events did not exhibit temporal relation with vaccination and were associated with other contributing factors, suggesting they may be coincidental. The identified causes for fetal death included idiopathic intrapartum asphyxia in 4 out of 6 cases and intrauterine growth retardation in 2 out of 6 cases. No temporal relation with vaccination was found in any of them, and half of the cases were linked to gestational hypertension.

Table 3 presents the proportions of pregnancy-related and fetal or neonatal events identified in the ESPIRTA study. This table includes both more common events (occurring in over 10% of cases) and common events (occurring in over 1% of cases), or those deemed significant for maternal–fetal health and therefore warranting special attention. Four pregnancy related events were included: cesarean section, gestational hypertension, premature rupture of membranes and gestational diabetes. Additionally, six fetal or neonatal events were also included: pre-term birth (gestational age < 37 weeks), low birth weight, small for gestational age, neonatal infection, congenital anomalies and stillbirth cases ≥ 20 weeks). Table 3 also presents the same events, recorded from the statistics of the five maternity hospitals utilized as historical reference.

**Table 3:** Proportions of pregnancy related and fetal or neonatal events in ESPIRTA study and hospital statistics

EVENT	ESPIRTA Study n/N (%)	Hospital Statistics 2020 * n/N (%)
Cesarean section	225/940 (23.94%)	5683/15839 (35.88%)
Gestational hypertension	67/940 (7.13%)	1811/12806 (14.14%)
PROM	65/940 (6.91%)	992/12806 (7.74%)
Gestational diabetes	48/940 (5.11%)	1578/12806 (12.32%)
Preterm birth (< 37 weeks)	72/949 (7.58%)	1105/15710 (7.75%)
Low birth weight	52/949 (5.48%)	973/15710 (6.19%)
SGA	38/949 (4.00%)	523/12729 (4.11%)
Neonatal infection	11/949 (1.16%)	129/12729 (1.01%)
Congenital anomalies	7/949 (0.74%)	33/1336 (2.47%)
Stillbirth ≥ 20 weeks	6/949 (0.63%)	234/15710 (1.49%)

In ESPIRTA study obstetric events were evaluated in 940 pregnant women. Events on fetal and neonatal events were evaluated in 949 cases

*PROM* Premature rupture of membranes, *SGA* Intrauterine growth retardation or small for gestational age

*2020 statistics from the five maternity hospitals participating in the study, as historical reference

Furthermore, the temporal relation between the events and vaccination was examined, in addition to assessing the frequency and severity of the events. The distribution of the events according to the dates of occurrence and vaccination was consistent across all subgroups of events (maternal morbidity, obstetric pathology, pathologies of ovular annexes, and fetal and neonatal events), Only 12 events were recorded within 48 h following the administration of any of the three doses in the primary immunization schedule or the booster dose. The events were: preterm birth threat (1 case), premature rupture of membranes (2 cases), pelvic presentation and low birth weight (both in the same participant), gestational hypertension (1 case), chronic hypertension (1 case), intrapartum fever (1 case), cesarean section (1 case) gastrointestinal infection (1 case). Three events were reported in newborns: neonatal icterus (2 cases) and risk for the well-being of the fetus (1 case). The occurrence of those events that had a close temporal relation to the vaccination could be explained by alternative causes.

### Immune response

#### Immune response according to SARS-CoV-2 infection

The presence or absence of maternal SARS-CoV-2 infection was taken into consideration for the analyses of the immunogenicity in terms of anti-RBD IgG antibodies in maternal and umbilical cord blood samples blood and IgA antibody titers in colostrum. Table 4 summarizes the results in those determinations and also shows PTR results. Overall, high anti-RBD IgG titers were detected in 189 maternal and 231 umbilical cord blood samples, with GMT of 1,392.15 (95% CI 1,174; 1,651) and 1,923 (95% CI 1,625; 2,275) respectively. There was a significant strong lineal correlation of Pearson between maternal and cord blood anti-RBD IgG titers (*r* = 0.7664, 95% CI 0.6982; 0.8208, *p* = 0.00). In 17 pregnant women infected by SARS-CoV-2 after vaccination higher antibody levels were found when compared with 172 non-infected women, without significant differences (*p* = 0.05). However, in the umbilical cord blood of 19 newborns from infected mothers significantly higher antibody levels were found (GMT of 4,231, CI95% 2,714; 6,596) when compared with 212 newborns from non-infected mothers (GMT of 1,792 95% CI 1,502; 2,137), *p* = 0.007. The placental transfer ratio (PTR) of anti-RBD IgG antibodies was determined in 179 mother–child dyads. In 71.5% of them (128 out of the 179) it was ≥ 1. The PTR had a median of 1.54 (IQR 1.48), indicating effective transfer. In the group of dyads positive for SARS-CoV-2, PTR was 1.54 (IQR 1.42) and 1.83 (IQR 1.46) in the negative ones. In both groups, PTR levels were ≥ 1, without statistical differences (*p* = 0.5). Anti-RBD IgA titers were determined in 197 samples from colostrum with global GMT levels of 1,227 (95% CI 986; 1,527). Although higher GMT were observed in the cases positives for SARS-CoV-2 infection (1,467, 95% CI 843; 2,553) in comparison with the non- infected group (1,208, 95% CI 956; 1,526), no significant differences were found (*p* = 0.6).

**Table 4:** Immunogenicity results according to maternal status regarding SARS-CoV-2 infection

Variable	SARS-CoV-2 infection		Global	*p*-value
	No	Yes		
Anti-RBD IgG antibodies in maternal blood	*N* = 172/228	*N* = 17/21	*N* = 189/249	0.5
GMT (95% CI)	1,353 (1,132; 1,618)	1,848 (997; 3,428)	1,392 (1,174; 1,651)	
Media ± SD	2,486 ± 3,026	4,149 ± 7,461	2,636 ± 3,646	
Median (IQR)	1,410 (2,365)	1,578 (1,973)	1,423 (2,345)	
(Min; Max)	(27; 17,916)	(209; 30,474)	(27; 30,474)	
N.D*	56	4	60	
Anti-RBD IgG antibodies in umbilical cord	*N* = 212/233	*N* = 19/21	*N* = 231/254	0.007
GMT (95% CI)	1,792 (1,502; 2,137)	4,231 (2,714; 6,596)	1,923 (1,625; 2,275)	
Media ± SD	3,552 ± 4,645	6,623 ± 7,578	3,805 ± 5,001	
Median (IQR)	1,948 (2,931)	3,009 (5,796)	2,036 (3,133)	
(Min; Max)	(35; 30,950)	(1,224; 31,251)	(35; 31,251)	
N.D*	21	2	23	
IgA titers in colostrum	*N* = 181/228	*N* = 16/21	197/249	0.6
GMT (95% CI)	1,208 (956; 1,526)	1,467 (843; 2,553)	1,227 (986; 1,527)	
Media ± SD	3,722 ± 7,837	2,909 ± 5,357	3,656 ± 7,658	
Median (IQR)	1,056 (2,119)	1,335 (1,598)	1,066 (2,114)	
(Min; Max)	(1; 49,862)	(434; 22,471)	(1; 49,862)	
N.D*	47	5	52	
Placental Transfer Ratio	*N* = 164/233	*N* = 15/21	*N* = 179/254	0.5
Media ± SD	2.05 ± 2.89	2.07 ± 1.69	2.05 ± 2.81	
Median (IQR)	1.54 (1.42)	1.83 (1.46)	1.54 (1.48)	
(Min; Max)	(0.09; 25.12)	(0.65; 7.42)	(0.09; 25.12)	
N.D*	69	6	75	

*GMT* Geometric mean titers, *95% CI* 95% confidence interval, *PTR* Placental transfer ratio, *SD* Standard deviation, *IQR* Interquartile rank, *Min*, *Max* Minimum, Maximum

Analyses were carried out according to Wilcoxon rank sum test at significance level of *p* < 0.05. * N.D: not determined

#### Immune response according to gestational age at birth

According to the gestational age at birth, in 203 newborns at term (gestational age ≥ 37 weeks) GMT of anti-RBD IgG titers were significantly higher 2.192 (95% CI 1,854, 2,592) in comparison with GMT in the 28 pre-term newborns (< 37 weeks), 743 (95% CI 413, 1,337), *p* < 0.001. The median of PTR at delivery was 1.58 (IQR 1.48) in 156 newborns birth at term and 0.92 (IQR 1.10) in 23 pre-term newborns, with significant differences between both groups, *p* = 0.02.

#### Immune response according to vaccination

When the immune response was evaluated according to the vaccination trimester in which the first dose of the immunization schedule was applied (including preconception, trimesters I, II, and III), no significant differences were found in any of the comparisons conducted. Considering the elapsed time between the last dose of the immunization schedule and delivery, the GMT (95% CI) of anti-RBD antibodies in maternal blood samples were 1,761 (95% CI 1,392; 2,229) in the participants that received the last dose less than 16 weeks before delivery and 1,134 (95% CI 891; 1,443) in women that received the last dose 16 weeks and more before delivery, *p* = 0.035. Significantly higher GMT in umbilical cord were also found in newborns form mothers that received the last dose of the immunization schedule less than 16 weeks before delivery 2,801 (CI 95% 2,232; 3,516) in comparison with newborns from women that received the third dose 16 weeks and more before delivery 1,413 (95% CI 1,121; 1,781), *p* < 0.001. No significant differences were found in the samples of colostrum and in the PTR values.

The immunogenicity results regarding the application, or not, of a booster dose showed that anti-RBD anti IgG antibodies, both in maternal and umbilical cord blood samples, were significantly higher in the participants that received a booster dose in addition to the primary immunization schedule. GMT in maternal samples was 1,784 (95% CI 1,416; 2,247) in women which received booster dose and 1,392 (95% CI 1,174; 1,651) in women that did not receive it, *p* = 0.021. In the respectively umbilical cord samples, GMT were 2,801 (95% CI 2,232; 3,516) and 1,413 (95% CI 1,121; 1,781), *p* < 0.001. No significant differences were found in the samples of colostrum and in the PTR values.

We evaluated the effect of the time since the last vaccine dose to delivery (where immunogenic assessment was carried out) and the PTR ≥ 1. In the logistic regression analysis, no influence of the time variable on the PTR was detected, OR 1.00 (95% CI 0.97; 1.03, *p* > 0.9). Pearson correlation indicated that there was not statistically significant association between the variables (*p* = 0.1895). In subgroup analyses, based on whether a booster dose was administered, a statistically significant association was observed between time since the last dose and delivery and the PTR ≥ 1 (OR 0.71, 95% IC 0.65; 0.77, *p* < 0.001). The mean time (IQR) between the last dose and delivery in subgroups that received booster dose was 9 weeks (IQR 10) and 21 weeks (IQR 8) in those women which only received the primary immunization schedule.

The detection of antibodies may be influenced by various factors; therefore, control variables that could interfere with the interpretation of the results and adverse events identified during the course of gestation, delivery, puerperium and those presented in the product of gestation were taken into account. A logistic regression model was utilized, selected using a backward selection procedure with the AKAIKE Information Criterion (AIC), which is used to compare different possible models and identify the one that best fits the data. The best-fit model, as determined according to AIC, was selected from all possible models which influenced whether the PTR ≥ 1. Only cesarean section exhibited a significant effect associated with a PTR > 1 (OR 0.30, 95% CI 0.13; 0.67, *p* = 0.003). The nature of this effect was negative, indicating that the PTR was less likely to be greater than 1 when a cesarean section was indicated.

#### Neutralizing antibodies for D614G and Omicron (B.1.1.529) variants and transfer across the placenta

**Fig. 2:**
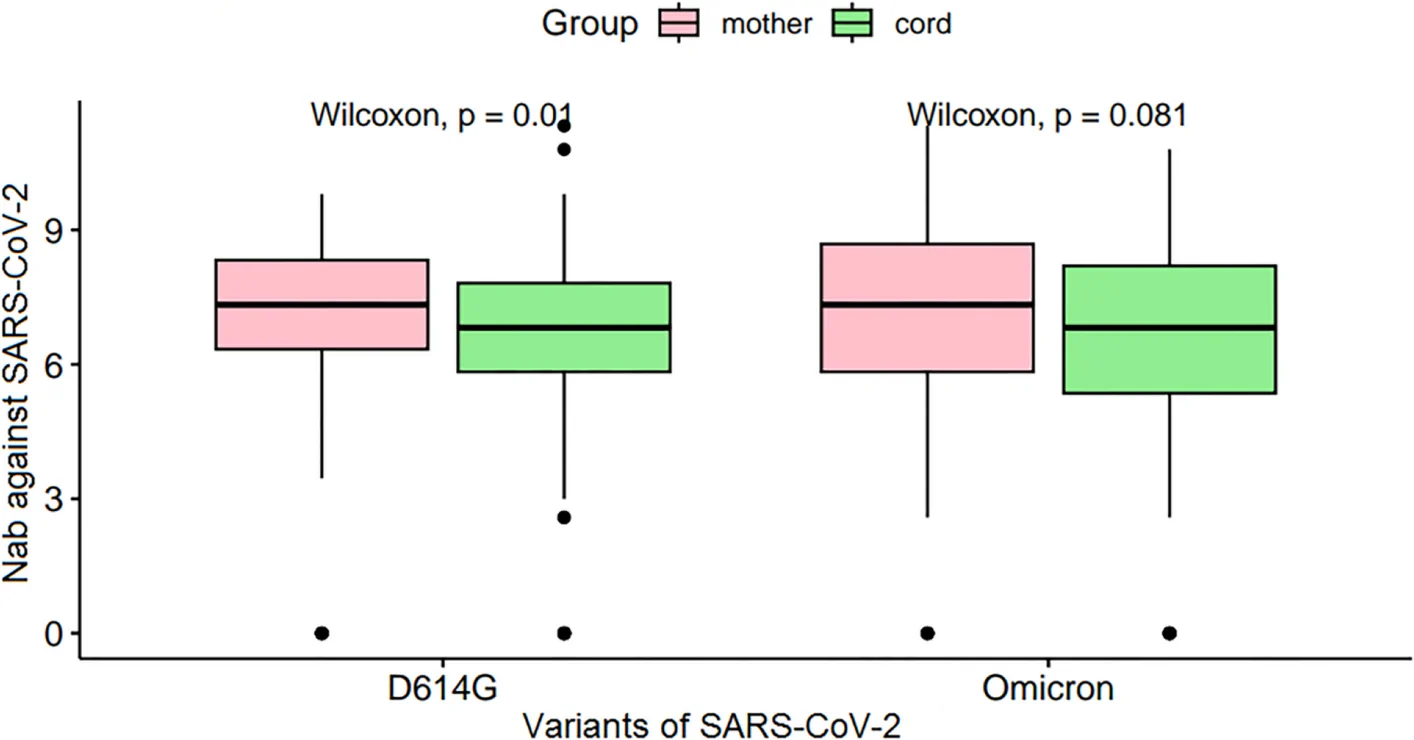
Neutralizing antibodies against D614G and Omicron strains of SARS-CoV-2 in maternal and umbilical cord blood. Legend: Nab: neutralizing antibodies, Omicron: B.1.1.529 strain

**Fig. 3:**
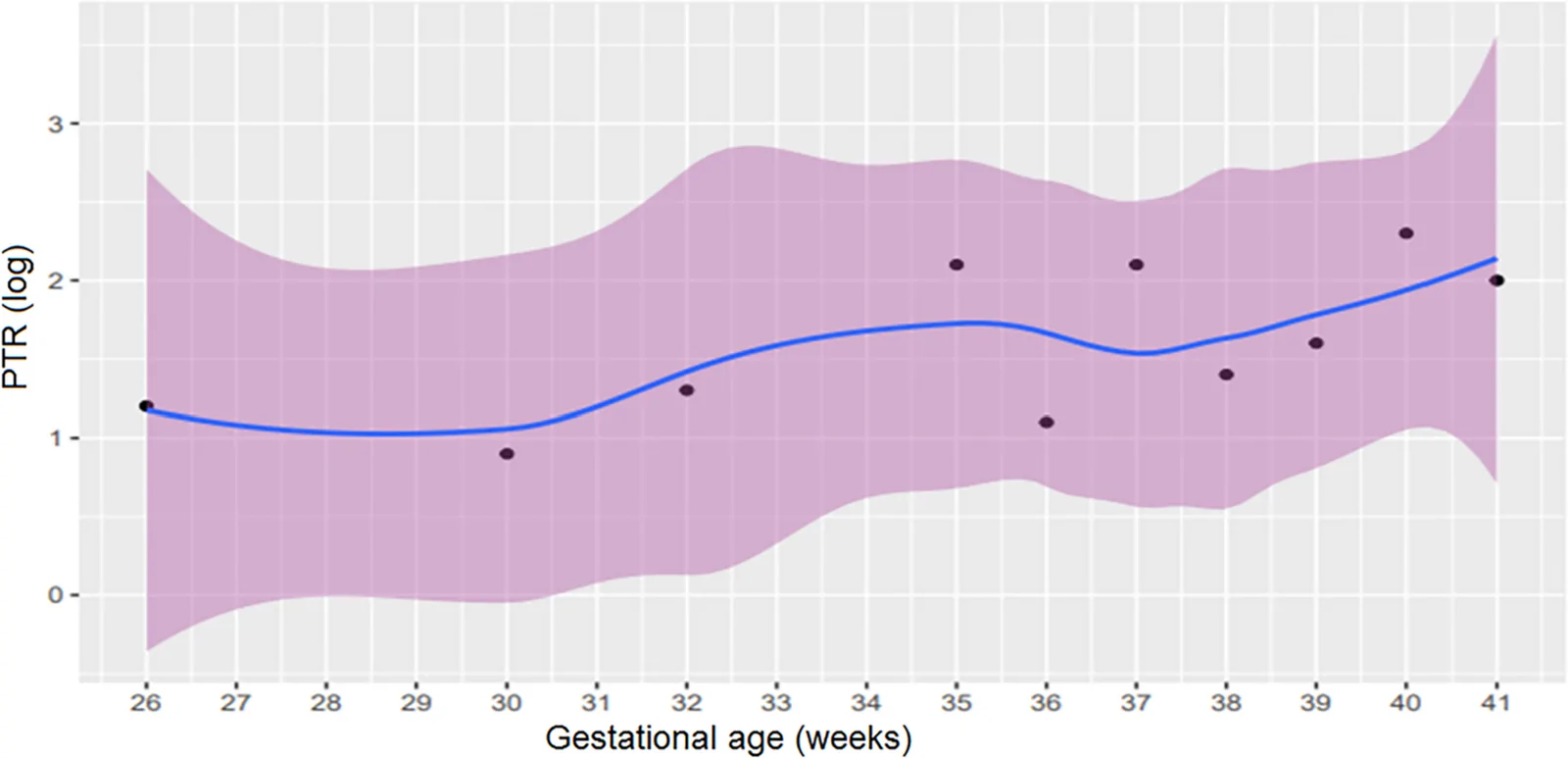
Placental transfer ratios according to gestational age at birth. Legend: PTR: placental transfer ratio. The black points represent the average of the PTR at each gestational age. The blue line means a smoothed line of the average with its 95% CI (violet band)

**Fig. 4:**
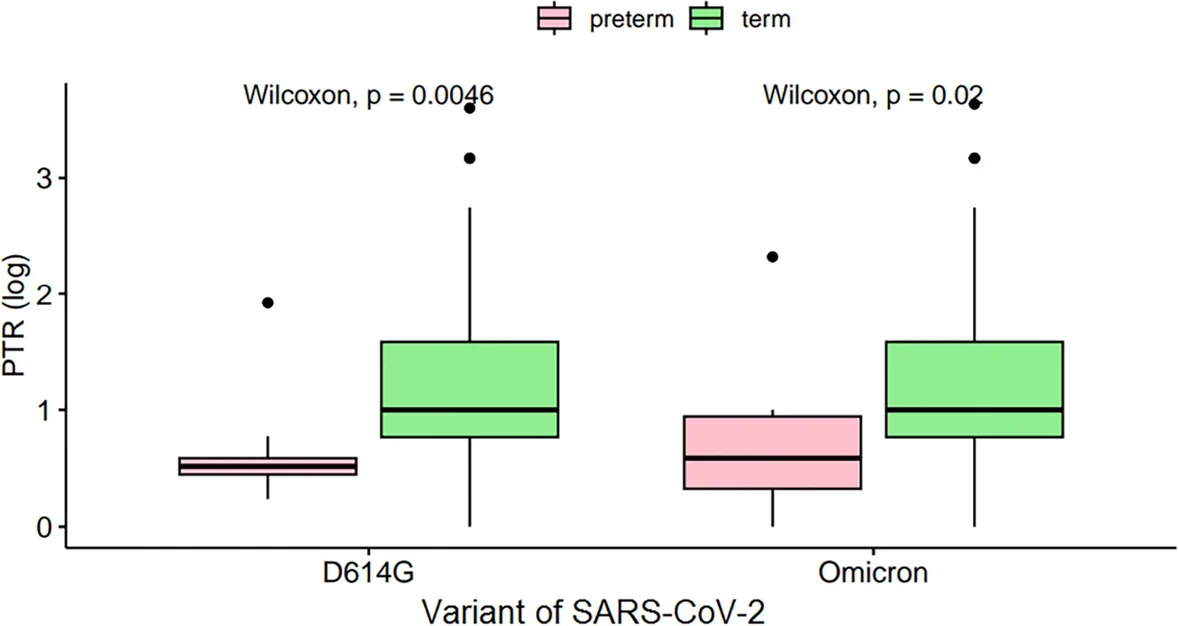
Placental Transfer Ratio of neutralizing antibodies against SARS-CoV-2 in term and preterm newborns. Legend: PTR: placental transfer ratio, Omicron: B.1.1.529 strain

**Fig. 5:**
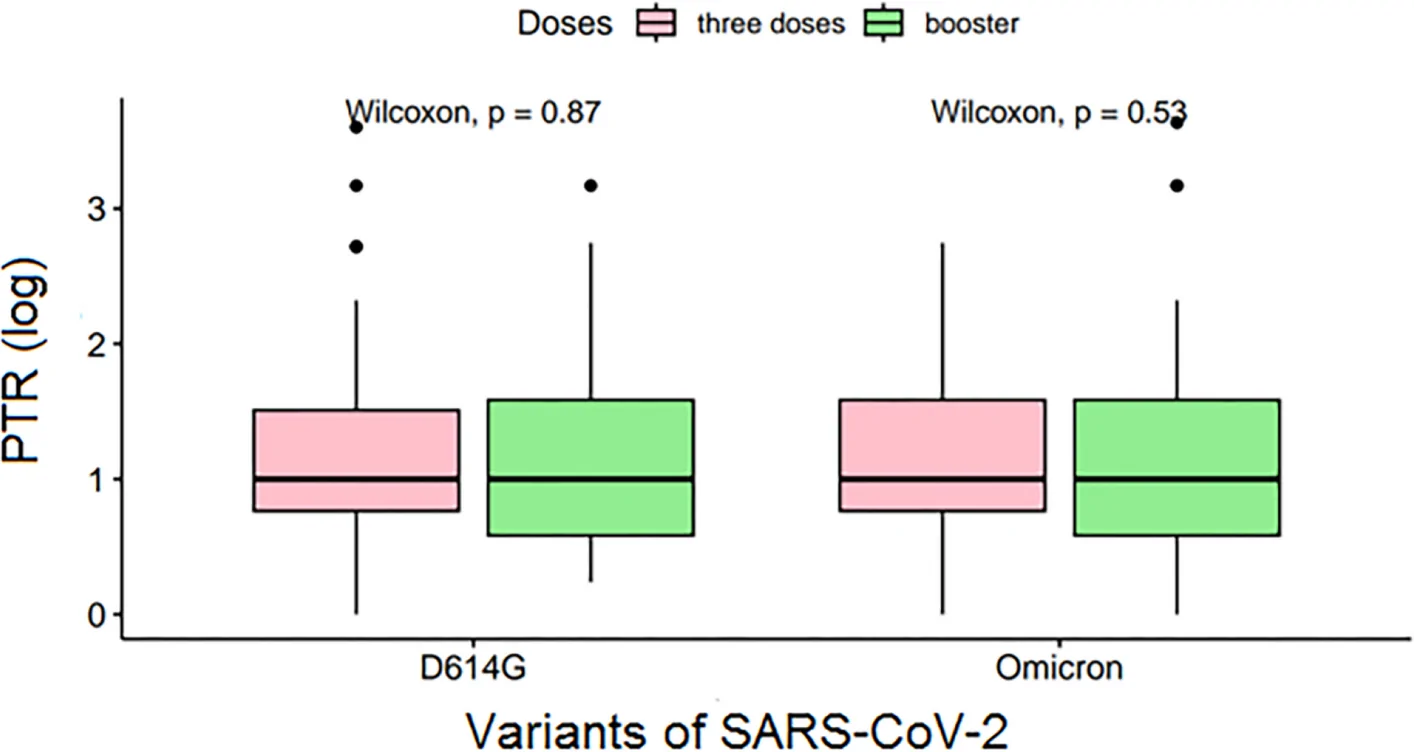
Placental transfer ratio according to number of doses received. Legend: PTR: placental transfer ratio, Omicron: B.1.1.529 strain

Neutralizing antibody (Nab) titers were evaluated for both D614G and Omicron (B.1.1.529) strains in 110 maternal-neonatal dyads. For D614G strain the Mean ± SD in maternal blood was 168.47 ± 2.96 and 172.15 ± 3.41 in umbilical cord. The median (IQR) was 1.00 (11.08). For Omicron B.1.1.529 strain the Mean ± SD in maternal blood was 113.82 ± 3.20 and 120.88 ± 3.88 in umbilical cord. The median (IQR) was 1.00 (1.30). Figure 2 shows the logarithmic expression of Nab titers in maternal blood and umbilical cord and the results of the comparisons according to Wilcoxon sum rank test. For D614G stain significant differences between maternal and umbilical cord were found, without significant differences relative to the Omicron strain. In both cases high PTR of Nab were achieved. Transfer of Nab titers across the placenta increased as gestational age advances (Fig. 3). PTR of Nab titers for D614G and Omicron (B.1.1.529) variants resulted significantly higher in neonates at term (gestational age > 37 weeks) regarding the preterm newborns (Fig. 4). No differences were found between mothers that received additional booster doses in comparison with those that only received the primary vaccination schedule (Fig. 5). PTR increased according to trimester of vaccination and when it occurred in the third trimester of pregnancy higher PTR were found although no significant differences were detected (*p* = 0.709, ANOVA test).

## Discussion

The ESPIRTA study was a multicenter, single-cohort observational investigation conducted to evaluate the safety and immunogenicity of the Abdala vaccine administered during pregnancy. This design was deemed the most suitable following the completion of the anti-COVID-19 vaccination campaign for all pregnant women, considering an epidemiological context where there was an increase in morbidity among pregnant women attributed to SARS-CoV-2 infection during the second half of 2021. While including a concurrent unvaccinated comparison group was not feasible, the study offers valuable real-world insights regarding the safety and immunogenicity of the Abdala vaccine given during pregnancy and to neonates. Given that bias is an inherent risk in observational studies, various strategies were employed in this research to reduce the biases typically linked to observational designs, thereby enhancing the reliability of the findings.

As noted before, information regarding AEFI and events throughout pregnancy were collected retrospectively. Recall bias has been described as a common limitation in retrospective studies in which participants are asked to remember prior behaviors or exposure, where the information that rely on patient memory, may be inaccurate or incomplete, especially regarding symptoms, or exposure histories. To reduce the impact of recall bias, current best practices emphasize reliance on objective data sources. In ESPIRTA study, we used properly documented sources that contributed to minimize inaccurate recall of past events or experiences. Criteria for participant selection, including inclusion and exclusion criteria, were established in the study protocol to guarantee precise information of the vaccination process (recorded on the vaccination card) and of medical events occurring after vaccination (documented in medical records and obstetric cards used for routine pregnancy follow-up). Altogether, those elements contributed to increase the internal validity of the study.

Since incorrect or inadequate exposure assessment can lead to measurement and misclassification bias, a key aspect of the study was the clear definition of exposure to the Abdala vaccine during pregnancy, which was verified from the vaccination card at inclusion. Although this strategy was important for reducing measurement and misclassification bias, it also increased the number of non-inclusions, which overall exceeded 60% of screened women, approximately half of them among women without documented exposure to the Abdala vaccine. For that reason, the possibility for selection bias remained difficult to exclude, particularly given the high proportion of screened women who were not included in the final cohort due to unavailable vaccination documentation.

It is worth emphasizing that the vaccination campaign to pregnant women, conducted by the Cuban Ministry of Public Health in July–August 2021, was carried out with Abdala, the only anti COVID-19 vaccine with an EAU in the country at that time. In ESPIRTA study, the exclusion criterion due to the unavailability of a vaccination card, and therefore the inability to confirm appropriate exposure to the Abdala vaccine, was only a requisite for the research purposes, and did not reflect differences regarding previous exposure to the vaccines during the vaccination campaign that occurred six months before the inclusion of the participants in the study. That exclusion criterion did not introduce differences between women included in the research and those non included, in terms of health care utilization or access to medical care during pregnancy, delivery, the puerperium and neonatal care. On the other hand, the exclusion criterion was not based on demographic or baseline characteristics, or on educational or socioeconomic factors, or any medical condition of the participants, which could otherwise, introduced differences from the target population and thereby affected the assessment of study outcomes. In addition, ESPIRTA was also a multicenter study conducted in five maternity hospitals in Havana, which, in principle, would contribute to the generalization of the results. Nevertheless, while the influence of this bias may be subject to debate, based on various factors previously mentioned, it may affect the generalizability of the study results to the broader population of pregnant women that received Abdala vaccine.

As it was mentioned before, all immunogenicity analyses were carried out only with participants from “Ramón González Coro” maternity hospital, which was the main clinical site of the study. The decision was taken for logistical reason in the context of the pandemic situation, to make more affordable the evaluation of a high number of samples, by different techniques. This strategy could raise the concern about bias selection and the generalizability of the results. As for safety evaluation, this subgroup of women from that hospital, were not selected based on any particular difference form the rest of the targeted population in ESPIRTA study that could explain differences regarding immunological results.

When ESPIRTA study started, background information was available through the detailed information regarding safety evaluation in controlled clinical trials in adults [10–12]. children and adolescents [13, 14]. It should be also note that COVID-19 subunit vaccines have been considered very safe, in accordance with this technological platform [19]. AEFI collected in the first 72 h after the application of Abdala vaccine in pregnant women in ESPIRTA study, had been previously reported for this vaccine in clinical trials and massive campaigns in non-pregnant population. They were common injection site and systemic events, that had a close temporal relationship to the vaccination and were attributable. Overall, the majority of these events were of mild intensity, and serious events did not occur.

Regarding maternal morbidity, SARS-CoV-2 infection was the common event most reported in our study. Interestingly, it was found that bronchial asthma significantly increased the risk of symptomatic infection. The results reported in a comparative analysis of symptomatic and asymptomatic COVID-19 carried out in 500 Qatari pregnant women, indicated that symptomatic women had 2.7 times higher odds of being asthmatic (adjusted OR 2.67, 95% CI 1.1, 6.7) [20].

The analysis of the incidence pregnancy related adverse events identified in the ESPIRTA study presented challenges, particularly since the vaccine was assessed in a cohort of pregnant women for the first time, and it was not feasible to compare the findings with a concurrent control group that did not receive the vaccine. In the context of the observational study design, it was not feasible to evaluate the identified events in relation to time-at-risk and population-at-risk from the available dataset. To provide contextual data from our settings as a reference for descriptive analyses, statistical information concerning some pregnancy-related and fetal-neonatal events from the same five maternity hospitals in the year 2020 was utilized (historical data). We utilized data on the evaluation of other anti-COVID-19 vaccines from the scientific literature to supplement the analyses of ESPIRTA study.

In a cohort of 35,691 pregnant women who received mRNA vaccines, no significant safety concerns were identified. The reported frequency of important events such as preterm birth (before 37 weeks) was 9.4%, small size for gestational age (3.2%) and major congenital anomalies (2.2%) [21]. In other study carried out in 1450 pregnant women an overall proportion of congenital malformations was 0.81% and 0.83% among pregnant exposed to the COVID-19 vaccine during the first and second/third trimester of pregnancy, respectively [22]. In ESPIRTA study, seven cases of congenital anomalies were identified, representing a frequency of 0.74%, while historical data from the five maternity hospitals indicated a frequency of 2.7% for this event.

A matched case–control study conducted within the Vaccine Safety Datalink investigated the relationship between COVID-19 vaccination and stillbirth. The study included 276 confirmed antepartum stillbirths and 822 live births, and it found no association between COVID-19 vaccination during pregnancy and stillbirth for two mRNA vaccines [23]. In ESPIRTA study, the frequency of stillbirths occurring at or beyond 20 weeks of gestation was recorded at 0.63%, with approximately half of these cases related with gestational hypertension. Hypertensive disorders in pregnancy are leading causes of maternal, fetal and neonatal morbidity and mortality worldwide. A large population-based study involving 135,466 pregnancies confirmed and quantified the magnitude of excess risk of small for gestational age and stillbirth among births to women with hypertensive disease during pregnancy [24].

A systematic review and meta-analysis investigating the safety of COVID-19 vaccines during pregnancy found no safety concerns associated with the currently administered vaccines. It is noteworthy that this conclusion primarily pertains to mRNA vaccines, as a significant majority of the pregnant participants in the study received this type of vaccine, particularly in high-income countries. Additionally, the review indicated no significant correlation between COVID-19 vaccination and adverse pregnancy outcomes, such as spontaneous abortion or congenital malformations, as assessed through comparative analyses of vaccinated and unvaccinated cohorts [25]. A systematic review examined the safety of COVID-19 vaccination during pregnancy, encompassing sixty-three studies, primarily retrospective observational and cohort studies, across eight different vaccine brands and various technological types, including mRNA, adenovirus vectors, and inactivated vaccines. Many studies focused on populations that received mRNA vaccines like Pfizer-BioNTech and Moderna. The analyses covered several factors, including preterm birth, stillbirth, birth weight, neonatal death, injection-site pain, fever, headache, and fatigue, among others. The review suggests that there are no severe adverse associated with COVID-19 vaccination in pregnant women, and mild effects, such as fever, were observed after COVID-19 vaccination in pregnant women [26].

The ESPIRTA study characterized the immune response in a cohort of pregnant women who received the Abdala vaccine during pregnancy. The results indicated elevated anti-RBD IgG titers in both maternal serum and cord blood samples, demonstrating a positive correlation between these values. The study also revealed an efficient transfer of IgG antibodies across the placenta, as well as the presence of anti-IgA antibodies in colostrum. Furthermore, neutralizing antibodies against two SARS-CoV-2 strains, specifically D614G and the more recent Omicron B.1.1.529 variant, were identified. Other studies concerning mRNA vaccines have indicated the presence of antibody transfer through the placenta and breast milk, suggesting a possible protective effect for neonates [27].

In a prospective cohort study involving 131 reproductive-age vaccine recipients, mRNA vaccines demonstrated strong humoral immunity in both pregnant and lactating women, exhibiting immunogenicity and reactogenicity comparable to that observed in non-pregnant women. Immune transfer to neonates occurred through both the placenta and breast milk [28]. Additionally, a study analyzing 122 pregnant women with available cord blood at the time of delivery revealed efficient antibody transfer across the placenta following maternal mRNA vaccination [29].

To achieve a more comprehensive characterization of the immunogenicity results from the ESPIRTA study, several factors were considered: the occurrence of SARS-CoV-2 infection post-vaccination, gestational age at birth, vaccination trimester, and time elapsed from vaccination to delivery, and having received booster doses. A longitudinal study involving 165 pregnant women, who were categorized into three cohorts based on their infection and vaccination status, revealed that breakthrough infections in vaccinated participants resulted in a significantly higher maternal RBD-specific IgG response compared to cases of either infection or vaccination alone, measured in both maternal plasma and breast milk. The cohort experiencing breakthrough infections exhibited the highest newborn-to-maternal ratio of RBD-specific IgG, demonstrating a strong correlation between maternal and newborn delivery titers. Breakthrough infections during pregnancy appear to extend pre-existing humoral immunity and facilitate trans placental antibody transfer to the neonate, reflecting an antibody priming effect attributable to prior vaccination rather than infection alone [30]. In ESPIRTA study, we compared the immunogenicity of pregnant women who experienced breakthrough infections after receiving any dose of the Abdala vaccine against those who were not infected. The breakthrough infection group exhibited elevated responses, notably in terms of anti-RBD IgG antibodies in both maternal serum and umbilical cord blood, as well as IgA antibodies in colostrum and PTR, particularly highlighting the significant differences in anti-RBD IgG antibodies detected in the umbilical cord.

In ESPIRTA study, significantly higher levels of anti-RBD IgG antibodies in umbilical cord blood and PTR were found in newborns birth at term (gestational age ≥ 37 weeks) compared to the levels found in the group of premature infants (gestational age < than 37 weeks). It has been previously reported that the transfer of immunoglobulin G (IgG) from the mother to the fetus begins at approximately 13 weeks of gestation. This transfer occurs progressively and linearly throughout the pregnancy, with the highest volume of IgG being transferred during the third trimester [31]. It has also been shown a consistent rise in IgG levels within the fetal circulation from 17 to 41 weeks of gestation. It is noteworthy that the majority of IgG is acquired by the fetus during the final four weeks of pregnancy [32]. In the ESPIRTA study, the transfer of antibodies through the placenta increased according the increase of gestational age. Furthermore, the placental transfer ratio (PTR) was significantly elevated in full-term neonates compared to those born prematurely. Similar findings were noted in the analyses of the PTR of neutralizing antibodies between term and preterm newborns for both D614G and Omicron B.1.1.529 strains of SARS-CoV-2.

In the logistic regression model applied using a backward selection procedure with the AKAIKE Information Criterion (AIC), the best-fit model identified by AIC indicated that cesarean section was the sole factor significantly associated with a propensity for the PTR to be greater than 1. The likelihood of a PTR exceeding 1 was reduced when a cesarean section was deemed necessary. Given that the occurrence of cesarean sections can be attributed to various factors, we did not find a plausible explanation for this finding, necessitating further analysis.

The antibody response is affected by additional or “booster” dose administration, the trimester time of vaccine administration, and the time passed from the first or second vaccine dose [33]. A study assessing how the timing of vaccination during pregnancy influences the placental transfer of antibodies revealed that vaccinations administered in the third trimester resulted in a greater transfer of antibodies compared to vaccination in the second trimester [34]. The ESPIRTA study yielded comparable PTR values from individuals vaccinated during the first and second trimesters. Additionally, a trend towards higher values was noted for vaccinations occurring in the third trimester, although these differences were not statistically significant. This lack of significance may be related to the sample size, as only 3% of participants received their vaccinations during the third trimester.

In a prospective cohort study involving 240 participants who received mRNA vaccines during pregnancy, it was observed that booster vaccination led to significantly higher binding and Nab titers, including those directed against the Omicron BA.1 variant, in maternal serum at delivery as well as in cord blood compared to a primary 2-dose series. The administration of a booster dose during pregnancy significantly increased maternal and cord blood binding and neutralizing antibody levels, including against Omicron BA.1 [35]. In the ESPIRTA study significantly higher levels of anti-RBD IgG were detected in both maternal and umbilical cord blood in the group of pregnant women who received a booster dose, relative to those who received only the primary immunization schedule. Additionally, PTR of anti-RBD IgG antibodies were also more elevated in women that received booster dose, although these differences did not reach statistical significance. No differences were identified in PTR of Nab between the two groups of pregnant women and for both D614G and Omicron B.1.1.529 strains of SARS-CoV-2.

A research involving 20 pregnant women who received the mRNA vaccine demonstrated efficient placental transfer of IgG antibodies following maternal vaccination for SARS-CoV-2. A positive correlation was identified between maternal and cord blood antibody concentrations. Furthermore, maternal and neonatal antibody levels were directly correlated to the time elapsed since vaccination. It is noteworthy that all participants in this cohort were vaccinated during the third trimester [36]. In the ESPIRTA study, the immunological evaluation was conducted based on the time interval between the last dose of the immunization schedule and delivery. Anti-RBD IgG titers in maternal and cord blood were significantly higher when the interval was < 16 weeks in comparison with those women that received the third dose ≥ 16 weeks before delivery. The administration of a booster dose may provide advantages, as evidenced by the observation that pregnant women who received a booster dose had a median time to delivery of 9 weeks, in contrast to those who did not receive it and had their last dose of the immunization schedule administered 24 weeks prior to delivery.

The measurement of IgA antibodies in colostrum samples within the ESPIRTA study was particularly valuable, given that the majority of published studies in the scientific literature have primarily concentrated on maternal serum and umbilical cord blood analyses. Human colostrum is recognized as a rich source of immune mediators that contribute to the immune defenses of newborns. Furthermore, colostrum is a good source of potentially bioactive molecules which support neonatal development and systemic lactogenic immune transfer of immunoglobulins that assists in the maintenance of innate immune defense [37].

In the ESPIRTA study, elevated levels of anti-IgA were identified in the colostrum, indicating a potential advantage that mothers vaccinated with the Abdala vaccine may confer to their newborns. It is essential to highlight the report of an investigation that assessed immune responses against SARS-CoV-2 spike RBD-specific IgA and IgG antibodies in breast milk following the administration of three doses of the protein subunit vaccine Abdala, specifically at intervals of 5 and 9 weeks post-vaccination. A cohort of 155 women was included, consisting of 103 vaccinated lactating mothers who had no prior COVID-19 infection and 52 unvaccinated women who had recovered from the disease. The study compared antibody levels from vaccinated women to those found in naturally immunized women after SARS-CoV-2 infection. Anti-RBD-specific IgA antibodies present in breast milk were expected to perform its neutralization function in the mucosa, which implies a potential passive protection received by breastfed infants from their mothers vaccinated with Abdala [38].

It should be noted that the ESPIRTA study was conducted between December 2021 and June 2022, including a cohort of 940 pregnant women who had received the Abdala vaccine as part of a health intervention in July–August 2021. In Cuba, although co-circulation of different predominant variants was observed in all epidemic waves; the Delta variant became predominant since June 2021. By September 2021, 100% of the samples analyzed at Cuban's National SARS-CoV-2 Reference Laboratory (Institute of Tropical Medicine Pedro Kourí, IPK) were classified as Delta, displacing the other circulating variants in the country and leading to increased morbidity and mortality from COVID-19 [39]. The vaccination campaign targeting pregnant and lactating women occurred during the most severe period of the pandemic within the country. In December 2021, coinciding with the commencement of the ESPIRTA study, the first case of COVID-19 attributed to the Omicron variant was detected in Cuba, shortly after the World Health Organization had classified Omicron as a variant of concern (VOC). Similar to trends observed in other countries, Omicron emerged when Delta while Delta was still the predominant variant and quickly replaced it. The peak of the first wave of Omicron in Cuba occurred during epidemiological weeks 2 to 4 in 2022. The Cuban wave was less intense compared to other countries, including those with high vaccination coverages. While case numbers also increased, the case fatality rates did not increase proportionally [40]. Thus, the ESPIRTA study, conducted from December 2021 to June 2022, took place during a period marked by the circulation of Omicron strains. Consequently, the cross-neutralizing neutralizing antibody (Nab) response against the Omicron VOC observed in serum samples from both mothers and newborns in the ESPIRTA study is of considerable interest. Our findings demonstrate that the PTR of neutralizing antibodies was elevated, exceeding 1.0 for both the D614G and Omicron variants, and no significant differences were identified between the two variants.

It is important to note that existing literature on the safety of COVID-19 vaccines in pregnant women primarily focuses on mRNA vaccines derived from studies conducted in high-income countries. For this reason, the ESPIRTA study provides valuable insights regarding the administration of a protein subunit Covid-19 vaccine to pregnant women in a low-income context, thereby augmenting the overall understanding of anti-COVID-19 vaccines utilizing this technological platform. The study investigated clinical relevant questions concerning the safety of Abdala vaccine, through pregnancy, delivery and the puerperium. Moreover, it assessed safety outcomes for both the fetus and newborns. The evaluation of immunogenicity in maternal serum, umbilical cord blood, and the colostrum, along with the measurement of neutralizing antibodies against two strains of SARS-CoV-2 and the placental transfer of antibodies from mother to neonates, represents strength of the study. The ESPIRTA study has some limitations. The observational design of the study, along with the absence of a concurrent control group comprising non-vaccinated pregnant women, restricts the ability to conduct definitive analyses to establish causality for adverse events. Given this observational framework, it was not feasible to evaluate the identified pregnancy-specific related events concerning time-at-risk and population-at-risk alignment from the available dataset, which is crucial for the interpretation of such outcomes. Statistical data regarding certain pregnancy-related and fetal-neonatal events from the same five maternity hospitals in the year 2020 were utilized as historical information solely to provide contextual data, as a reference for descriptive analyses. However, this historical data did not constitute a properly matched group for comparing adverse events frequency, thereby limiting the interpretability of the results and preventing robust inferences regarding the absence of excess risk. Despite several measures implemented to reduce potential biases, the interpretation of safety must be contextualized within the observational nature of the study and its inherent limitations. The potential for selection bias cannot be dismissed, particularly given the high proportion of screened women who were excluded from the final cohort, due to the lack of unavailable vaccination documentation necessary for ensuring accurate information regarding vaccine exposure. This limitation may affect the generalizability of the results to the broader population of pregnant women who received the Abdala vaccine.

## Conclusions

The ESPIRTA study provides valuable information concerning the application of the Abdala vaccine in specific populations, such as pregnant women, which was not available prior to this study. The safety evaluation of the Abdala vaccine during pregnancy, delivery and the puerperium, as well as in fetus-newborn, revealed no safety signals, as indicated by this cohort study. Elevated anti-RBD IgG titers were detected, in both in maternal serum and cord samples, indicating a positive correlation between them. Moreover, an efficient transfer of IgG antibodies across the placenta was demonstrated. The high anti-IgA titers found in the colostrum may provide an additional advantage regarding the passive transfer of antibodies from mother to newborn through breastfeeding. Furthermore, neutralizing antibodies against two SARS-CoV-2 strains, D614 G and the more recent Omicron B.1.1.529 variant, were identified. Further research is recommended to assess the long-term safety and efficacy of the Abdala vaccine for pregnant women and their newborns.

## Supplementary Information

Supplementary Material 1.

## Data Availability

Anonymised participant data will be made available when the trial is complete, upon requests directed to the corresponding author ([verena.muzio@cigb.edu.cu](mailto:verena.muzio@cigb.edu.cu)). Proposals will be reviewed and approved by the sponsor, researcher, and collaborators on the basis of scientific merit. After approval of a proposal, data can be shared through a secure online platform after signing a data access agreement.
