# Supplementary material for: Assessment of safety and immunogenicity of a protein subunit COVID-19 vaccine (ABDALA) in pregnant women: a single-cohort, multicenter, observational ESPIRTA study
Source: BMC Pregnancy Childbirth. 2026 Jun 24;26:998. doi: 10.1186/s12884-026-09488-1 (PMC13551808; doi:10.1186/s12884-026-09488-1)
Supplement: Supplementary file 1 [file 12884_2026_9488_MOESM1_ESM.docx]

**Other Investigators (ESPIRTA Research Group):**

**“Ramón González Coro" Maternal Hospital, Havana:** Susana Cuenca Osorio MD, Karen Muñoz Ros BSc., Mayte Trujillo Ricaño MD, Liuba Núñez Fernández BSc., Dayami Ajete Machado BSc., Ibis Castillo de la Nuez MD, Beatriz de la Cruz Roldán MD, Camilo Andrés Anaya Herazo MD, Penélope Alessandra Tome MD, Sheyla Barcelona Reyes MD, Sililay Fajardo Reyes MD, Verena Muzio Rivas MD, Rogelio González Sánchez MD, Julio Borrego López MD.

**“Eusebio Hernandez” Maternal Hospital”, Havana:** Verónica Valdez Echevarría MD, Nahyla Lilliet Armenteros BSc., Patricia Pedroso Hernández MD, Aurelia Maria Peñalver Cruz MD.

**“America Arias” Maternal Hospital“, Havana:** Dayana Martin Terrero MD, Arlety Suarez López.

**“Genetic Engineering and Biotechnology Centre”, Havana:** Elizeth García Iglesias BSc., Julio Esmir Baldomero Hernández MD, Hugo Nodarse Cuni, PhD., Ricardo Ulises Martínez Rosales BSc., Yahima Chacón Quintero BSc., Sheila Chavez Valdes BSc., Giselle Freyre Corrales BSc., Edelgis Coizeau Rodriguez Tech.
